# Exploring the Conceptual Constructs of Learners’ Goal Commitment, Grit, and Self-Efficacy

**DOI:** 10.3389/fpsyg.2021.783400

**Published:** 2021-10-29

**Authors:** Zhong Han

**Affiliations:** College of Marxism, Weifang University of Science and Technology, Weifang, China

**Keywords:** learners’ goal commitment, learners’ grit, learners’ self-efficacy, learning achievement, passion and perseverance

## Abstract

Although learners’ success in learning has generally been attributed to cognitive factors, non-cognitive issues in education should be taken into consideration in the process of learning which affects learners’ achievement. One of these issues, which become popular among researchers in the previous decade is grit, that is, posited as passion and perseverance thanks to its enduring quality and the other is self-efficacy. Another factor is goal commitment that talks about the way to reach a goal or insistent determinations to achieve a goal. The proposed review attempts to focus on these three factors in regulating students’ learning achievement. Accordingly, some educational suggestions are offered for teachers, students, and syllabus designers.

## Introduction

Educators and instructive counselors have attempted to recognize and comprehend the fundamental aspects that bring about the achievement and failure of learners during the past decades (Vergara, 2020). The learning cycle is a continuous practice that includes an effort to endure, particularly despite hardships and disadvantages (Binning et al., 2018). Experimental studies confirm the significance of qualities other than intellectual capacity (Levin, 2013; Dweck et al., 2014; Heckman et al., 2014). Several non-cognitive factors in the instructive field, like self-assurance, grit, and self-efficacy, can bring about the ideal learning result (Alhadabi and Karpinski, 2020). Indeed, constructive features like grit and self-efficacy have been viewed among the most essential predictors of achievement in people (Miller and Kass, 2019). As learners perform better in a classroom setting, grit and self-efficacy have significant roles in their success. Learners’ success is positively affected when educators can handle daily classroom activities, address learners’ individual needs, and establish meaningful connections with them (Aloe et al., 2014; Zee and Koomen, 2016; Troesch and Bauer, 2017). Self-efficacy and grit share the trait of allowing an individual to cope with difficulty and persist despite obstacles. Excitement, motivation, effort, and optimism are also considered to be part of grit (Miller and Kass, 2019).

Indeed, self-efficacy is viewed as an eminent non-cognitive element, established in the social cognitive hypothesis developed by a notable psychologist of the 20th century known as Albert Bandura. Individuals’ conviction regarding their capacity to succeed is known as self-efficacy. Individuals who consider themselves as well-organized attribute accomplishment to individual effort, while those with a low degree of efficacy ascribe it to external factors (Fathi and Derakhshan, 2019; Fathi et al., 2021). On the one hand, individuals with a high degree of efficiency ascribe potential failures to a lack of individual effort. On the other hand, individuals who view themselves as inefficient, ascribe failure to their low capacities and capabilities (Malureanu et al., 2021). Based on Bandura’s concept, it can be understood that belief in one’s capacities leads to one perceiving assignments with more courage, being able to conquer them easily without any problems, which results in stress reduction and a decrease in the risk of nervousness (Malureanu et al., 2021). Self-efficacy (the person’s assessment of his or her abilities) is a powerful indicator of educational performance, academic success, and many other key factors (Chemers et al., 2001; Feldman and Kubota, 2015; Fathi et al., 2020). Self-efficacy can be characterized as one’s convictions regarding their ability to deliver specified degrees of performance that have an impression on events influencing their lives (Bandura, 2010). The term alludes to believing in one’s aptitude to accomplish and execute the blueprints needed to reach success. Accordingly, self-efficacy alludes to one’s view of their capacity to carry out an assignment or to take part in the action (Zwart et al., 2020). Self-efficacy is connected to the feeling of self-assurance in one’s capacity and can be firmly identified with the interest to learn more or the craving to participate in assignments that are perceived as difficulties in contrast to assignments of general interest (Han and Wang, 2021; Malureanu et al., 2021). Mohammadyari (2012) stated that self-efficacy is directly associated with how much energy is spent in the learning process, perseverance, the kinds of tasks learners perform, and subsequently their performance. Honicke and Broadbent (2016) found a correlation between self-efficacy and academic success. When an individual is self-efficacious, he or she sets more ambitious targets, takes on greater risk, commits to their objectives more firmly, and strives to achieve them (Luszczynska et al., 2005).

Moreover, grit is regarded as a predecessor to self-efficacy (Usher et al., 2019) and it has been shown that learners with grit, persistence, and enthusiasm for long-term success, achieve more in education and health outcomes (Guerrero et al., 2016; Datu et al., 2017). The latest research has proved that grit, as motivation and commitment toward the accomplishment of long-term ambitions, plays a significant role in academic performance and educational success (Eskreis-Winkler et al., 2014). Grit can be characterized as acting enthusiastically and perseveringly to attain long-term objectives despite hardships (Duckworth et al., 2007). Therefore, individuals with more prominent degrees of grit are more committed when attempting to overcome hindrances. Moreover, they maintain their interest to accomplish their objectives regardless of failures, challenges, and/or absence of help (Arslan et al., 2013). The developing body of literature portrays grit’s relationship with scholarly, individual, and behavioral paradigms.

A significant element of grit is goal-setting, which strives for longstanding objectives and it plays a noteworthy role in every aspect of life, and one of the most important prerequisites for setting goals is to be motivated to learn. Consequently, motivated learners are tended to view their education as a process toward attaining their aims and put forth an effort to accomplish this (Zivanovic and Subotin, 2018). The goal-setting theory holds that when people are motivated to achieve their objectives, the association between goal and behavior (accomplishment) is the most influential (Locke and Latham, 2002). Commitment to achieving goals is necessary for motivation because goals cannot be accomplished without effort (Human-Vogel and Rabe, 2015). As well as a moral obligation, commitment is also a cognitive obligation that demonstrates responsibility and dedication to a particular purpose (Klein et al., 2012).

Commitment alludes to emotional factors including attentiveness, confidence, and acknowledgment of positive perspectives toward specific things (Kim and Ok, 2009). The general impression, fulfillment, sense of belonging, insight of value, and appreciation for a specific foundation are called institutional commitment (Meyer and Allen, 2004). Commitment is an issue of learners’ perseverance in higher education (Strauss and Volkwein, 2004). To emphasize the important effect of commitment on the quest for the objective, Oettingen et al. (2009) characterized it as a prerequisite for objective accomplishment. Burkley et al. (2013) demonstrated that people with a high level of commitment exert more energy for objective fulfillment and devote more determination and effort, and are consequently bound to succeed. To emphasize the important effect of commitment on the quest for the objective, Oettingen et al. (2009) characterized it as a requirement for objective accomplishment. Burkley et al. (2013) demonstrated that people with a high level of commitment exert more energy for objective fulfillment and allocate more determination, and are consequently bound to flourish (Fishbach et al., 2006).

Grounded on the review of literature, the development of learners’ success and their capability to preserve a constructive and dynamic setting is based on three important theories, namely self-determination theory by Deci and Ryan (1985), Bandura’s self-efficacy theory (Bandura, 1997), and grit theory by Duckworth et al. (2009). Although each variable was examined before, there is a paucity of literature about the relationship of these three constructs in learning contexts. To this end, the current review endeavors to inspect the role of learners’ goal commitment, grit, and self-efficacy.

## Review of Literature

### Self-Efficacy

As asserted by Miller (2011), the idea of self-efficacy arisen from Bandura’s original work in the social learning hypothesis, and within that hypothesis, Bandura portrayed learning as acquiring information intellectually through the processing of data attained by observing others. As stated by Miller (2011), the social learning hypothesis identifies three associated learning factors, namely, mental qualities, conduct, and environment. Through his work with the social learning hypothesis, Bandura started to notice that one’s feeling of achievement and capacity to proceed despite challenging assignments assumed a critical part in learning. This led to Bandura developing the self-efficacy hypothesis. Self-efficacy is a strong factor in deciding how an individual will act, think, and respond when confronted with difficult circumstances. It is fundamental in developing learners’ character to enhance their studying process (Thompson and Verdino, 2019). The probability of success is enhanced when faced with obstacles and failures if a learner has high self-efficacy (Van Dinther et al., 2011). To develop emotional self-regulation skills, they must not become stressed and should have self-control so that they are not distressed with apprehension (Bandura, 2010). Self-efficacy is a dominant issue in defining how an individual performs, thinks, and reacts in case of challenging circumstances (Alavi et al., 2017; Downes et al., 2017). Self-efficacy is crucial in evolving learners’ behavior to simplify their learning progression (Fan and Williams, 2010; Roddenberry and Renk, 2010; Van Dinther et al., 2011). Nonetheless, regarding scholarly functioning, self-efficacy levels allude to discrepancies across diverse degrees of assignments, like increasing the intricacy of math questions. Generalization relates to the transmission of self-efficacy convictions across exercises, like diverse scholastic topics. Levels of conviction that one can perform given assignments indicates the strength of perceived efficacy (Brouwer et al., 2010). Students with high self-efficacy are likely to make insistent determinations, conscientiousness, persistence, and grit (Lightsey et al., 2011; Raqshin and Nirjar, 2012; Datu et al., 2017). A typical trait of these learners is a desire to achieve excellent results, a higher enthusiasm toward learning, extensive reading, and researching, not easily disappointed, and view failure positively (Al Mutir, 2015; Shikalepoh, 2016). The ability to adapt to new circumstances and demands is enhanced when one has a higher sense of self-efficacy (Axford, 2007; Seifalain and Derakhshan, 2018; Han and Wang, 2021).

### Grit

Seligman and Csikszentmihalyi (2000) have provided a great deal to the advancement of constructive behavior as the end of the 20th century (MacIntyre et al., 2019; Wang et al., 2021) by encouraging different issues such as enthusiasm, motivation, and grit. Grit is portrayed through hard work in managing difficulties, maintaining exertion and interest over the years despite being confronted with failure, resilience, and challenges (Duckworth et al., 2007). Furthermore, grit echoes a mental variable based on positive psychology, which focused on persistence as a marker of long-term achievement and is related to accomplishing high-level objectives for long period (Von Culin et al., 2014; Duckworth, 2016). Furthermore, Grit is one of the qualities that assist a person with changing the perception that the determinant of progress is just knowledge. It portrays how one can accomplish long-term objectives by overwhelming hindrances and difficulties. In addition, it is one way to figure out, where one can invest their energy to survive when confronting difficulties in life (Hochanadel and Finamore, 2015). Duckworth (2016) divided grit into two parts, namely passion and perseverance. Each one has both a single and aggregate impact on an individual’s ability to develop and maintain grit. Grit has been acknowledged as a major predictor of success among individuals during the past two decades (Duckworth et al., 2007; Maddi et al., 2012; Strayhorn, 2014). Perseverance is observed when people persist and keep moving forward despite difficult circumstances, failures, or resistance. Learners develop grit by establishing a system for managing and moving past mistakes and rejections over time (Duckworth, 2016). Students learn how to distinguish between low-level and high-level targets and decide where to use their efforts. According to Duckworth (2016), grit is not a result of intelligence, but rather the eagerness to learn and grow through one’s enthusiasm for a particular activity. According to Duckworth (2016), putting effort into people’s skills allows them to develop faster. A hard-working mindset and a sense of commitment are traits of grittier people as they approach life as a test of endurance. People may experience challenges and hardships along the way, but grit implies that they can remain focused and keep striving until they reach their goal (Duckworth et al., 2007; Robertson-Kraft and Duckworth, 2014).

### Goal Commitment

One’s perseverance to accomplish a specified objective is known as goal commitment (Oettingen et al., 2009; Burkley et al., 2013). Researchers have stated that commitment to accomplishing an objective is what prompts an individual throughout the whole objective cycle to the achievement of that objective (Burkley et al., 2013; Mann et al., 2013). However, laying out a particular objective at the beginning does not mean one is committed enough to make a plan and act according to it to accomplish that objective. Since goal commitment is significant across the stages of the objective cycle, researchers have endeavored to distinguish the factors that decide why certain individuals are more committed to their objectives than others. How people regard their objective was the main focus of most researchers. For example, studies originating from the expectancy-value hypothesis have inspected the functions that objective expectancy (i.e., the perceived probability of objective achievement) and objective worth (i.e., perceived advantages of objective accomplishment) have in deciding goal commitment during the three general stages of the objective cycle (Brandstätter and Frank, 2002; Sun et al., 2014).

## Implications and Future Directions

This review attempts to focus on the thoughtful facet of three important factors in learning progress such as grit, self-efficacy, and goal commitment. The review of literature elucidates the prominence of non-cognitive factors in the route of learning. Centered on the review of literature, it can be clinched that students with high self-efficacy have greater objectives and are more dedicated to accomplishing them. Developing people’s ambitions, choosing specific actions, applying a great deal of effort, and having persistence despite challenges are the results of their belief that they can succeed (Raqshin and Nirjar, 2012). Grit plays a major role in the achievement of objectives since it motivates learners to adhere to their plans and to be committed and perseverant in goal setting (Duckworth, 2016) and it is one of the characteristics that can enable a learner to persevere in challenging circumstances, and it is enhanced by self-efficacy and it enhances commitment to a significant objective despite the disappointment. When a learner believes that they can accomplish their objectives with grit, their self-efficacy encourages them (Siah et al., 2019). Engaging educators in a discussion and creating a collaborative environment around grit factors can support learners’ achievement. These courses should target learners directly as well since they are the principal agents of success and development. It is suggested that goal commitment should be the target for improving grit so policymakers and experts should make efforts to promote grit by setting the goals. The goal-setting theory suggests that individuals with a commitment to their goals do their best to show perseverance in achieving their objective and the progress of goal commitment is indispensable for success in the learning process. The intervention function of grit indicates that goal commitment and grit can be directed as key facts for those anticipating to develop learners’ engagement, as well (Liu, 2021; Xie and Derakhshan, 2021).

In scholastic settings, learners who need scholarly self-efficacy cannot perform well, so they experience difficulty in continuing and being effective learners. Researchers have emphasized that the absence of one’s confidence in their capacity to overcome difficult assignments eventually influences their degree of motivation and perseverance when confronted with misfortune. Self-efficacious individuals tend to take on more complex responsibilities. So the implication of this study is for teachers to create more ambitious settings and strive to achieve them (Luszczynska et al., 2005), which results in making a quick decision in case of setbacks and being focused on their objectives. Teachers should encourage high degrees of efficacy in learners because learners with high degrees of self-efficacy work hard to learn and accomplish. They have an undeniable degree of commitment, goal, and their perseverance in undertaking hard tasks consequently convert into higher levels of accomplishment (Duckworth and Gross, 2014). Students’ self-efficacy is raised when they experience accomplishment by achieving the objectives they set. Students with low self-efficacy keep away from specific assignments or excuse themselves when they are not effective in accomplishing their objectives (Robbins et al., 2013). Moreover, by raising learners’ awareness regarding grit and through grit training, teachers can enhance learners’ abilities and can improve their self-confidence, and learners who have self-confidence are more probable to support their classmates in resolving difficulties and making decisions. If challenges and ambiguities are handled efficiently and effectively, learners are capable of accomplishing their objectives.

The consequence of the present review is for learners because by creating an environment that encourages self-awareness and grit, they can adopt more effective teaching methods and objectives, resulting in better learning achievement. High self-efficacy likewise permits them to choose challenging settings and investigate their current circumstances or make new ones. Therefore, it depicts confidence in their skill in managing a wide range of demands. According to Schunk et al. (2010), learners with objectives and a sense of self-efficacy for achieving them take part in exercises that they believe will prompt achievement and satisfaction. Students will probably analyze their performances and progress as they work on the assignment once they commit to endeavor for an objective. Self-assessments of progress raise self-efficacy and maintain motivation. Learners who have higher self-efficacy are likely to exert the necessary energy and persevere in the face of educational difficulties. As a result, findings demonstrate that self-efficacy influences educational performance (Schunk and Zimmerman, 2006).

Higher educational organizations should put effort into assisting their learners with fostering the necessary information, abilities, and skills. Even though, competent practice generally relies upon obtaining information and abilities, it is clear that learners’ self-efficacy plays an anticipating and intervening role concerning their accomplishments, motivation, and learning. Hence, it appears to be vital that administrators of higher education focus on learners’ development of self-efficacy. Knowing the elements that affect the improvement of learners’ self-efficacy can help higher education organizations in creating and arranging instructive projects that upgrade learners’ self-efficacy. Thus, creating such a learning setting that stimulates grit and self-efficacy can be a valued issue to be taken into account by faculty members’ instructional determinations.

According to Locke and Latham (2002), the objective-performance connection is greatest when individuals are profoundly dedicated to their objectives. Thus, regardless of whether an individual is viewed as gritty, their grit cannot add to their scholarly accomplishment if scholastic accomplishment is not their objective, or if their dedication to this objective is not strong. By creating an environment that encourages self-awareness and grit, learners can adopt more effective teaching methods and objectives, resulting in better learning achievement, and also by creating a supportive and autonomous educational setting, self-efficacy can be enhanced (Pekrun, 2006). So by developing learners’ self-efficacy and grit, faculty members can ensure their highest levels of success. The development of self-efficacy and goal commitment should help learners to improve their presentation.

## Conclusion

This conceptual analysis theoretically reviewed the three significant constructs in the non-cognitive issues in general education and this review posists that the non-cognitive variables should be given due attention in that they have also affected learners’ achievement. In particular, grit that embraces passion as well as perseverance, self-efficacy that is about determining how an individual will act and goal commitment that is mainly concerned with the pathway to achieve a goal have been theoretically documented. Lastly, this conceptual analysis also attempts to make some important implications for some stakeholders, teachers, students, and syllabus designers.

## Author Contributions

The author confirms being the sole contributor of this work and has approved it for publication.

## Conflict of Interest

The author declares that the research was conducted in the absence of any commercial or financial relationships that could be construed as a potential conflict of interest.

## Publisher’s Note

All claims expressed in this article are solely those of the authors and do not necessarily represent those of their affiliated organizations, or those of the publisher, the editors and the reviewers. Any product that may be evaluated in this article, or claim that may be made by its manufacturer, is not guaranteed or endorsed by the publisher.

## References

[ref1] Al Mutir A. (2015). Student Choice in Continuing to Study High School Science (Doctoral dissertation), Fredonia, New York.

[ref2] Alavi A. Zargham-Boroujeni A. Yousefy A. Bahrami M. (2017). Altruism, the values dimension of caring self-efficacy concept in Iranian pediatric nurses. J. Educ. Health Promot. 6, 1–5. doi: 10.4103/jehp.jehp_142_14, PMID: 28546973 PMC5433645

[ref3] Alhadabi A. Karpinski A. C. (2020). Grit, self-efficacy, achievement orientation goals, and academic performance in university students. Int. J. Adolesc. Youth 25, 519–535. doi: 10.1080/02673843.2019.1679202

[ref4] Aloe A. M. Amo L. C. Shanahan M. E. (2014). Classroom management, self-efficacy and burnout: a multivariate meta-analysis. Educ. Psychol. Rev. 26, 101–126. doi: 10.1007/s10648-013-9244-0

[ref5] Arslan S. Akin A. Citemel N. (2013). The predictive role of grit on metacognition in Turkish university students. Stud. Psychol. 55, 311–320. doi: 10.21909/sp.2013.04.645

[ref6] Axford K. M. (2007). Attachment, Affect Regulation, and Resilience in Undergraduate Students (Doctoral dissertation), Walden University, United States.

[ref7] Bandura A. (1997). Self-Efficacy: The Exercise of Control. New York, NY: W.H. Freeman and Company.

[ref8] Bandura A. (2010). “Self-efficacy” in The Corsini Encyclopedia of Psychology. eds. Weiner B. Craighead W. E. (New York: Wiley), 1534–1536.

[ref9] Binning K. R. Wang M. Te Amemiya J. (2018). Persistence mindset among adolescents: who benefits from the message that academic struggles are normal and temporary? J. Youth Adolesc. 48, 269–286. doi:10.1007/s10964-018-0933-3, PMID: 30276598

[ref10] Brandstätter V. Frank E. (2002). Effects of deliberative and implemental mindsets on persistence in goal-directed behavior. Personal. Soc. Psychol. Bull. 28, 1366–1378. doi: 10.1177/014616702236868

[ref11] Brouwer S. Reneman M. F. Bültmann U. Van der Klink J. J. Groothoff J. W. (2010). A prospective study of return to work across health conditions: perceived work attitude, self-efficacy and perceived social support. J. Occup. Rehabil. 20, 104–112. doi: 10.1007/s10926-009-9214-z, PMID: 19894106 PMC2832875

[ref12] Burkley E. Anderson D. Curtis J. Burkley M. (2013). Vicissitudes of goal commitment: satisfaction, investments, and alternatives. Pers. Individ. Differ. 54, 663–668. doi: 10.1016/j.paid.2012.11.033

[ref13] Chemers M. M. Hu L. T. Garcia B. F. (2001). Academic self-efficacy and first-year college student performance and adjustment. J. Educ. Psychol. 93, 55–64. doi: 10.1037/0022-0663.93.1.55

[ref14] Datu J. A. D. Yuen M. Chen G. (2017). Grit and determination: a review of the literature with implications for theory and research. J. Psychol. Couns. Sch. 27, 168–176. doi: 10.1017/jgc.2016.2

[ref15] Deci E. L. Ryan R. M. (1985). Intrinsic Motivation and Self-Determination in Human Behavior. New York, NY: Plenum.

[ref16] Downes P. E. Kristof-Brown A. L. Judge T. A. Darnold T. C. (2017). Motivational mechanisms of self-concordance theory: goal-specific efficacy and person–organization fit. J. Bus. Psychol. 32, 197–215. doi: 10.1007/s10869-016-9444-y

[ref17] Duckworth A. (2016). Grit: The Power of Passion and Perseverance. New York, NY: Scribner.

[ref18] Duckworth A. Gross J. (2014). Self-control and grit: related but separable determinants of success. Curr. Dir. Psychol. Sci. 23, 319–325. doi: 10.1177/0963721414541462, PMID: 26855479 PMC4737958

[ref19] Duckworth A. L. Peterson C. Matthews M. D. Kelly D. R. (2007). Grit: perseverance and passion for long-term goals. J. Pers. Soc. Psychol. 92, 1087–1101. doi: 10.1037/0022-3514.92.6.1087, PMID: 17547490

[ref20] Duckworth A. L. Quinn P. D. Seligman M. E. P. (2009). Positive predictors of teacher effectiveness. J. Posit. Psychol. 4, 540–547. doi: 10.1080/17439760903157232

[ref21] Dweck C. Walton G. Cohen G. (2014). Academic Tenacity: Mindsets and Skills that Promote Long-Term Learning. Seattle, WA: Bill & Melinda Gates Foundation.

[ref22] Eskreis-Winkler L. Duckworth A. L. Shulman E. P. Beal S. (2014). The grit effect: predicting retention in the military, the workplace, school and marriage. Front. Psychol. 5:36. 10.3389/fpsyg.2014.00036, PMID: 24550863 PMC3910317

[ref23] Fan W. Williams C. M. (2010). The effects of parental involvement on students’ academic self-efficacy, engagement and intrinsic motivation. Educ. Psychol. 30, 53–74. doi: 10.1080/01443410903353302

[ref24] Fathi J. Derakhshan A. (2019). Teacher self-efficacy and emotional regulation as predictors of teaching stress: an investigation of Iranian english language teachers. Teach. English Lang. 13, 117–143. doi: 10.22132/TEL.2019.95883

[ref25] Fathi J. Derakhshan A. Arabani A. S. (2020). Investigating a structural model of self-efficacy, collective efficacy, and psychological well-being among Iranian EFL teachers. Iranian J. Appl. Linguist. Stud. 12, 61–80. doi: 10.22111/IJALS.2020.5725

[ref26] Fathi J. Greenier V. Derakhshan A. (2021). Self-efficacy, reflection, and burnout among Iranian EFL teachers: the mediating role of emotion regulation. Iranian J. Lang. Teach. Res. 9, 13–37. doi: 10.30466/IJLTR.2021.121043

[ref27] Feldman D. B. Kubota M. (2015). Hope, self-efficacy, optimism, and academic achievement: distinguishing constructs and levels of specificity in predicting college grade-point average. Learn. Individ. Differ. 37, 210–216. doi: 10.1016/j.lindif.2014.11.022

[ref28] Fishbach A. Dhar R. Zhang Y. (2006). Subgoals as substitutes or complements: the role of goal accessibility. J. Pers. Soc. Psychol. 91, 232–242. doi: 10.1037/0022-3514.91.2.232, PMID: 16881761

[ref29] Guerrero L. R. Dudovitz R. Chung P. J. Dosanjh K. K. Wong M. D. (2016). Grit: a potential protective factor against substance use and other risk behaviors among Latino adolescents. Acad. Pediatr. 16, 275–281. doi: 10.1016/j.acap.2015.12.016, PMID: 26796576 PMC4821776

[ref30] Han Y. Wang Y. (2021). Investigating the correlation among Chinese EFL teachers’ self-efficacy, work engagement, and reflection. Front. Psychol. 12:763234. doi: 10.3389/fpsyg.2021.763234 PMC8603392 34803845

[ref31] Heckman J. J. Humphries J. E. Kautz T. (2014). The Myth of Achievement Tests: The GED and the Role of Character in American Life. Chicago: University of Chicago Press.

[ref32] Hochanadel A. Finamore D. (2015). Fixed and growth mindset in education and how grit helps students persist in the face of adversity. J. Int. Educ. Res. 11, 47–50. doi: 10.19030/jier.v11i1.9099

[ref33] Honicke T. Broadbent J. (2016). The influence of academic self-efficacy on academic performance: a systematic review. Educ. Res. Rev. 17, 63–84. doi: 10.1016/j.edurev.2015.11.002

[ref34] Human-Vogel S. Rabe P. (2015). Measuring self-differentiation and academic commitment in university students: a case study of education and engineering students. South Afr. J. Psychol. 45, 60–70. doi: 10.1177/0081246314548808

[ref35] Kim W. Ok C. (2009). The effects of relational benefits on customers’ perception of favorable inequity, affective commitment, and repurchase intention in full-service restaurants. J. Hosp. Tour. Res. 33, 227–244. doi: 10.1177/1096348008329874

[ref36] Klein H. J. Molloy J. C. Brinsfield C. T. (2012). Reconceptualizing workplace commitment to redress a stretched construct: revisiting assumptions and removing confounds. Acad. Manag. Rev. 37, 130–151. doi: 10.5465/amr.2010.0018

[ref37] Levin H. M. (2013). “The utility and need for incorporating noncognitive skills into large-scale educational assessments,” in The Role of International Large-Scale Assessments: Perspectives from Technology, Economy, and Educational Research. eds. Von Davier M. Gonzalez E. Kirsch I. Yamamoto K. (New York, NY: Springer), 67–86.

[ref38] Lightsey O. R. Maxwell D. A. Nash T. M. Rarey E. B. McKinney V. A. (2011). Self-control and self-efficacy for affect regulation as moderators of the negative affect–life satisfaction relationship. J. Cogn. Psychother. 25, 142–154. doi: 10.1891/0889-8391.25.2.142

[ref39] Liu J. (2021). The role of grit in students’ L2 engagement in the English as a foreign language classroom. Front. Psychol. 12:749844. doi: 10.3389/fpsyg.2021.749844, PMID: 34557136 PMC8452846

[ref40] Locke E. A. Latham G. P. (2002). Building a practically useful theory of goal setting and task motivation: a 35-year odyssey. Am. Psychol. 57, 705–717. doi: 10.1037/0003-066X.57.9.705, PMID: 12237980

[ref41] Luszczynska A. Gutiérrez-Doña B. Schwarzer R. (2005). General self-efficacy in various domains of human functioning: evidence from five countries. Int. J. Psychol. 40, 80–89. doi: 10.1080/00207590444000041

[ref42] MacIntyre P. D. Gregersen T. Mercer S. (2019). Setting an agenda for positive psychology in SLA: theory, practice, and research. Mod. Lang. J. 103, 262–274. doi: 10.1111/modl.12544

[ref43] Maddi S. R. Matthews M. D. Kelly D. R. Villarreal B. White M. (2012). The role of hardiness and grit in predicting performance and retention of USMA cadets. Mil. Psychol. 24, 19–28. doi: 10.1080/08995605.2012.639672

[ref44] Malureanu A. Panisoara G. Lazar I. (2021). The relationship between self-confidence, self-eefficacy, grit, usefulness, and ease of use of e-learning platforms in corporate training during the COVID-19 pandemic. Sustainability 13, 1–20. doi: 10.3390/su13126633

[ref45] Mann T. De Ridder D. Fujita K. (2013). Self-regulation of health behavior: social psychological approaches to goal setting and goal striving. Health Psychol. 32, 487–498. doi: 10.1037/a0028533, PMID: 23646832

[ref46] Meyer J. P. Allen N. J. (2004). TCM Employee Commitment Survey Academic Users Guide 2004. London, Ontario, Canada: The University of Western Ontario, Department of Psychology.

[ref47] Miller P. H. (2011). Theories of Developmental Psychology 5th *Edn*. New York: Worth Publishers.

[ref48] Miller E. Kass E. (2019). Enhancing self-efficacy and grit: How educational teams can promote inner strengths of students with disabilities in inclusive schools. In EDULEARN19 Proceedings 11th International Conference on Education and New Learning Technologies: Palma, Spain. July 1–3, 2019; IATED Academy. 2003–2011.

[ref49] Mohammadyari G. (2012). Comparative study of relationship between general perceived self-efficacy and test anxiety with academic achievement of male and female students. Procedia Soc. Behav. Sci. 69, 2119–2123. doi: 10.1016/j.sbspro.2012.12.175

[ref50] Oettingen G. Mayer D. Sevincer A. T. Stephens E. J. Pak H. J. Hagenah M. (2009). Mental contrasting and goal commitment: the mediating role of energization. Personal. Soc. Psychol. Bull. 35, 608–622. doi: 10.1177/0146167208330856, PMID: 19213924

[ref51] Pekrun R. (2006). The control-value theory of achievement emotions: assumptions, corollaries, and implications for educational research and practice. Educ. Psychol. Rev. 18, 315–341. doi: 10.1007/s10648-006-9029-9

[ref52] Raqshin S. Nirjar A. (2012). Accruing individual potential for creativity and innovation in biotechnology firms. Int. J. Innov. Learn. 11, 162–181. doi: 10.1504/IJIL.2012.045174

[ref53] Robbins S. P. Decenzo D. A. Coulter M. (2013). Fundamentals of Management: Essential Concepts and Applications. 8th *Edn*. New Jersey: Pearson Education.

[ref54] Robertson-Kraft C. Duckworth A. L. (2014). True grit: trait-level perseverance and passion for long-term goals predicts effectiveness and retention among novice teachers. Teach. Coll. Rec. 116, 1–27. PMID: 25364065 PMC4211426

[ref55] Roddenberry A. Renk K. (2010). Locus of control and self-efficacy: potential mediators of stress, illness, and utilization of health services in college students. Child Psychiatry Hum. Dev. 41, 353–370. doi: 10.1007/s10578-010-0173-6, PMID: 20204497

[ref56] Schunk D. H. Pintrich P. R. Mecee J. L. (2010). Motivation in Education: Theory, Research and Applications. New Jersey, NJ: Prentice Hall.

[ref57] Schunk D. H. Zimmerman B. J. (2006). “Competence and control beliefs: distinguishing the means and ends,” in Handbook of Educational Psychology. eds. Alexander P. A. Winne P. H. (Mahwah, NJ: Erlbaum), 349–367.

[ref58] Seifalain M. Derakhshan A. (2018). The relationship between Iranian EFL teachers’ burnout and self-efficacy across English-related vs. non-English-related academic degrees. Int. J. English Lang. Transl. Stud. 6, 99–110.

[ref59] Seligman M. E. P. Csikszentmihalyi M. (2000). Positive psychology: an introduction. Am. Psychol. 55, 5–14. doi: 10.1037/0003-066X.55.1.5 11392865

[ref60] Shikalepoh P. P. (2016). Learners’ Self-Efficacy Beliefs in Reading Comprehension in English Second Additional Language in a Namibian Rural School. Doctoral dissertation, North-West University (South Africa), Potchefstroom Campus.

[ref61] Siah P. C. Ng A. H. W. Dharmaraj E. Foo C. Tan S. M. Wider W. (2019). Grit personality as a mediator or moderator for the effects of internet addiction on procrastination. J. Institut. Res. South East Asia 17, 18–32.

[ref62] Strauss L. C. Volkwein J. F. (2004). Predictors of student commitment at two-year and four-year institutions. J. High. Educ. 75, 203–227. doi: 10.1353/jhe.2004.0007

[ref63] Strayhorn T. L. (2014). What role does grit play in the academic success of black male collegians at predominantly white institutions? J. Afr. Am. Stud. 18, 1–10. doi: 10.1007/s12111-012-9243-0

[ref64] Sun S. Vancouver J. B. Weinhardt J. M. (2014). Goal choices and planning: distinct expectancy and value effects in two goal processes. Organ. Behav. Hum. Decis. Process. 125, 220–233. doi: 10.1016/j.obhdp.2014.09.002

[ref65] Thompson K. V. Verdino J. (2019). An exploratory study of self-efficacy in community college students. Commun. Coll. J. Res. Pract. 43, 476–479. doi: 10.1080/10668926.2018.1504701

[ref66] Troesch L. M. Bauer C. E. (2017). Second career teachers: job satisfaction, job stress, and the role of self-efficacy. Teach. Teach. Educ. 67, 389–398. doi: 10.1016/j.tate.2017.07.006

[ref67] Usher E. L. Li C. R. Butz A. R. Rojas J. P. (2019). Perseverant grit and self-efficacy: are both essential for children’s academic success? J. Educ. Psychol. 111, 877–902. doi: 10.1037/edu0000324

[ref68] Van Dinther M. Dochy F. Segers M. (2011). Factors affecting students’ self-efficacy in higher education. Educ. Res. Rev. 6, 95–108. doi: 10.1016/j.edurev.2010.10.003

[ref69] Vergara C. R. (2020). Grit, self-efficacy and goal orientation: a correlation to achievement in statistics. Int. J. Educ. Res. 8, 89–106.

[ref70] Von Culin K. R. Tsukayama E. Duckworth A. L. (2014). Unpacking grit: motivational correlates of perseverance and passion for long-term goals. J. Posit. Psychol. 9, 306–312. doi: 10.1080/17439760.2014.898320, PMID: 31404261 PMC6688745

[ref71] Wang Y. L. Derakhshan A. Zhang L. J. (2021). Researching and practicing positive psychology in second/foreign language learning and teaching: the past, current status and future directions. Front. Psychol. 12:731721. doi: 10.3389/fpsyg.2021.731721, PMID: 34489835 PMC8417049

[ref72] Xie F. Derakhshan A. (2021). A conceptual review of positive teacher interpersonal communication behaviors in the instructional context. Front. Psychol. 12:708490. doi: 10.3389/fpsyg.2021.708490, PMID: 34335424 PMC8319622

[ref73] Zee M. Koomen M. Y. (2016). Teacher self-efficacy and its effects on classroom processes, student academic adjustment, and teacher well-being: a synthesis of 40 years of research. Rev. Educ. Res. 86, 981–1015. doi: 10.3102/0034654315626801

[ref74] Zivanovic A. M. Subotin M. D. (2018). The goal setting components: a study of Serbian EFL learners. Методички видици 9, 235–248. doi: 10.19090/mv.2018.9.235-248

[ref75] Zwart D. P. Noroozi O. Van Luit J. E. Goei S. L. Nieuwenhuis A. (2020). Effects of digital learning materials on nursing students’ mathematics learning, self-efficacy, and task value in vocational education. Nurse Educ. Pract. 44:102755. doi: 10.1016/j.nepr.2020.102755, PMID: 32199243

